# Case Report: Co-infection of tuberculosis and pertussis in a 47-day-old infant and therapeutic strategies

**DOI:** 10.3389/fped.2025.1673286

**Published:** 2025-11-18

**Authors:** Qingping Tang, Ruonan Li, Xiaotao Yang, Yanchun Wang, Yonghan Luo

**Affiliations:** 1 Second Department of Infectious Disease, Kunming Children's Hospital (Kunming Medical University affiliated), Kunming, Yunnan, China; 2 Faculty of Life Science and Technology, Kunming University of Science and Technology, Kunming, Yunnan, China

**Keywords:** infant, tuberculosis, pertussis, co-infection, drug-induced liver injury

## Abstract

**Background:**

Tuberculosis (TB) and pertussis are both highly contagious diseases caused by *Mycobacterium tuberculosis (M. tuberculosis)* and *Bordetella pertussis (B. pertussis)*, respectively, with significant morbidity and mortality among children. However, neonatal co-infection with these two pathogens is extremely rare, and no such cases have been reported in the literature to date.

**Case presentation:**

We reported a case of a 47-day-old full-term male infant admitted with cough and fever. Chest imaging revealed bilateral pulmonary consolidation. Bronchoalveolar lavage confirmed co-infection with *M. tuberculosis* and *B. pertussis*. Initial empirical antibiotic therapy was ineffective. Based on pathogen identification, treatment was adjusted to a combination of levofloxacin, isoniazid, rifampicin, and pyrazinamide. During treatment, the infant developed drug-induced liver injury, prompting several modifications to the anti-TB regimen, including the introduction of linezolid and adjunctive corticosteroids. With individualized therapeutic adjustments and close liver function monitoring, the infant's condition improved significantly, and he was eventually discharged in stable condition.

**Conclusion:**

This case underscores the importance of considering atypical pathogens in neonates presenting with severe unexplained pneumonia, particularly in regions endemic for TB and pertussis. Tailored antimicrobial strategies and dynamic assessment of drug toxicity, especially hepatotoxicity, are essential for successful management.

## Introduction

Tuberculosis (TB), caused by *Mycobacterium tuberculosis (M. tuberculosis)*, remains one of the leading causes of mortality worldwide (1). According to the World Health Organization, China ranks among the countries with the highest TB burden globally. Although the incidence of TB has declined in recent years, pediatric pulmonary TB continues to constitute a significant portion of the disease spectrum (2, 3). Neonatal tuberculosis, while rare, can result from either congenital infection or postnatal acquisition (4, 5). However, current research on the diagnosis and treatment of neonatal TB remains limited.

*Bordetella pertussis (B. pertussis)*, a Gram-negative bacillus, is another widespread pathogen of global concern. It is a major cause of infectious disease, characterized primarily by paroxysmal, spasmodic coughing, and is prevalent among children and adolescents (6, 7). In recent years, the incidence of pertussis has been steadily rising, with a concurrent increase in severe cases and mortality rates among young infants (8).

Due to developmental considerations, neonates face numerous pharmacological contraindications, particularly when infected with multiple distinct pathogens, complicating therapeutic decision-making. To our knowledge, no reports have yet documented cases of neonatal co-infection with tuberculosis and pertussis. Therefore, we present this case of a one-month-and-seventeen-day-old male infant diagnosed with concurrent TB and pertussis, aiming to provide clinical insight into the management of such complex infections.

**Figure 1:**
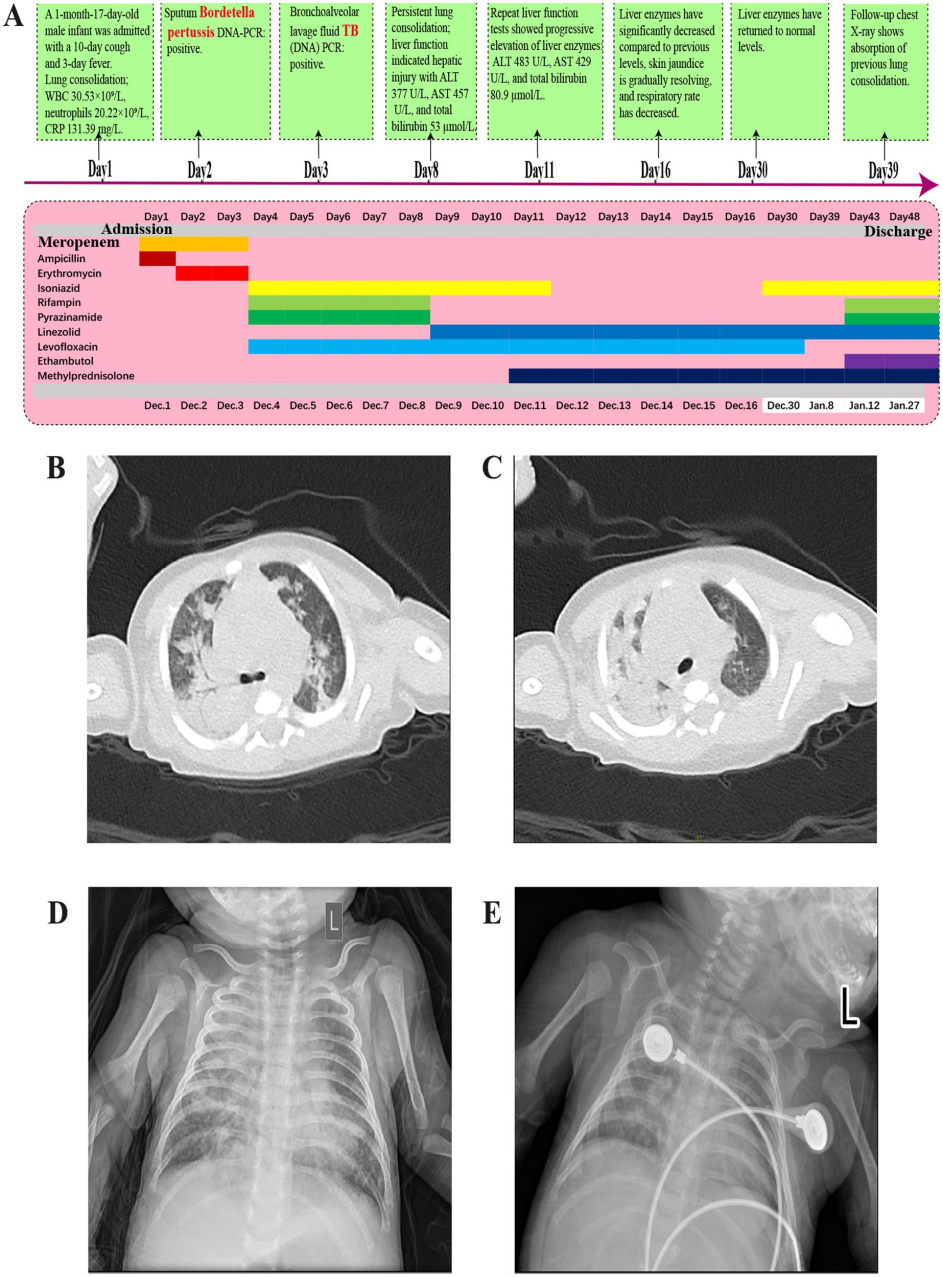
**(A)** Diagnostic and treatment timeline of the disease. **(B,C)** Chest CT showing bilateral pneumonia with consolidation and a small right-sided pleural effusion. **(D)** Chest x-ray after 5 days of anti-TB treatment showing pneumonia with partial consolidation. **(E)** Chest x-ray after 1 month of anti-TB treatment showing partial absorption of pneumonia and consolidation compared to before.

**Figure 2:**
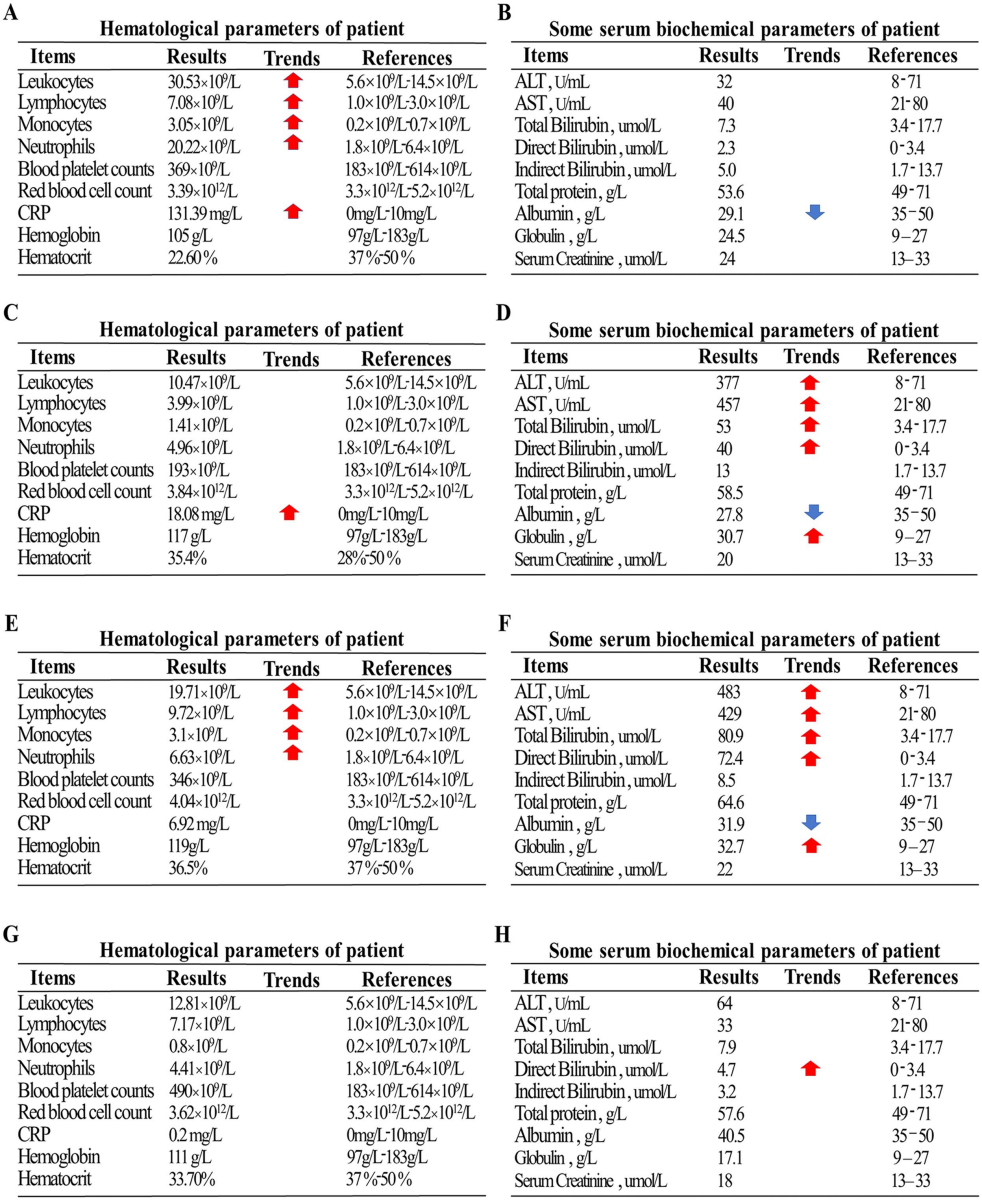
**(A)** Initial complete blood count on admission showing marked leukocytosis. **(B)** Initial liver function test on admission showing normal liver enzymes. **(C)** Blood count on day 5 of anti-TB treatment showing normalization of hematologic parameters. **(D)** Liver function test on day 5 of anti-TB treatment showing significant elevation of liver enzymes and increased bilirubin. **(E)** Blood count on day 3 after discontinuation of rifampicin showing a rise in white blood cells. **(F)** Liver function test on day 3 after discontinuation of rifampicin showing persistent elevation of liver enzymes and total bilirubin. **(G,H)** Final pre-discharge blood count and liver function tests, both within normal ranges.

A 47-day-old full-term male infant, delivered vaginally, who had received Bacillus Calmette–Guérin (BCG) vaccination at birth according to the national immunization schedule, was admitted with a ten-day history of coughing and a three-day history of fever. His mother had received BCG and diphtheria–tetanus–pertussis (DTP) vaccinations during childhood according to the national immunization program. Initially presenting with a mild cough, his symptoms progressively worsened, evolving into paroxysmal coughing. Three days prior to admission, he developed a fever with a peak temperature of 38.4°C. On admission, he exhibited tachypnea (respiratory rate 60 breaths/min), tachycardia (heart rate 160 bpm), significant hypoxia, and positive signs of chest retractions. Chest CT performed on the day of admission revealed bilateral pneumonia with pulmonary consolidation (Figures 1B,C). Laboratory tests showed leukocytosis with a WBC count of 30.53 × 10^9^/L, neutrophil count of 20.22 × 10^9^/L, and elevated C-reactive protein (CRP) at 131.39 mg/L (Figure 2A). Liver function was normal (Figure 2B). A bacterial pulmonary infection was suspected, and empiric antibiotic therapy with meropenem and ampicillin was initiated (Figure 1A).

On hospital day 2, a PCR test for *B. pertussis* DNA returned positive, leading to the substitution of ampicillin with erythromycin for targeted treatment.

On day 3, the patient underwent bronchoalveolar lavage, and PCR testing of the lavage fluid was positive for *M. tuberculosis* DNA, confirming a diagnosis of pulmonary tuberculosis co-infected with *B. pertussis*. In addition, a comprehensive pathogen screening was performed, encompassing respiratory syncytial virus, influenza virus, parainfluenza virus, adenovirus, rhinovirus, and Mycoplasma pneumoniae. Except for *B. pertussis* and *M. tuberculosis*, all other pathogen assays yielded negative results. Further testing, including GeneXpert analysis of the lavage fluid, indicated rifampicin-sensitive TB. Cerebrospinal fluid analysis was unremarkable. Epidemiologic investigation revealed that the infant's mother had experienced a persistent cough during pregnancy, and subsequent testing (sputum GeneXpert) confirmed rifampicin-sensitive pulmonary TB. She was admitted to a designated infectious disease hospital for treatment. Despite combined therapy with meropenem and erythromycin, the infant's condition did not improve. Considering the high prevalence of macrolide-resistant *B. pertussis* strains in China and the national guideline recommendation against sulfamethoxazole-trimethoprim use in infants under two months of age, and the presence of concurrent TB a modified regimen was initiated. The patient was started on intravenous levofloxacin, isoniazid, rifampicin, and pyrazinamide to cover both infections.

By day 8 (fifth day of anti-TB treatment), follow-up chest radiograph still showed significant pulmonary consolidation (Figure 1D). Liver function tests indicated hepatotoxicity with elevated ALT (377 U/L), AST (457 U/L), and total bilirubin (53 µmol/L), alongside visible jaundice (Figures 2C,D). Drug-induced liver injury from rifampicin and pyrazinamide was suspected, prompting discontinuation of both drugs and substitution with intravenous linezolid.

By day 11 (three days after stopping rifampicin and pyrazinamide), liver enzymes continued to rise (ALT 483 U/L, AST 429 U/L, total bilirubin 80.9 µmol/L), suggesting isoniazid-related hepatotoxicity (Figures 2E,F). Isoniazid was discontinued, and the patient was treated with linezolid and levofloxacin, alongside intravenous methylprednisolone (1 mg/kg/day) for inflammation control.

By day 16 (fifth day after isoniazid discontinuation), liver enzyme levels decreased significantly, jaundice resolved, and respiratory symptoms improved (reduced respiratory rate, alleviated paroxysmal cough). High-flow oxygen therapy was transitioned to nasal cannula, feeding tolerance improved, and weight gain was noted. By day 30, liver enzymes normalized, and isoniazid was reintroduced.

On day 39, chest radiograph (Figure 1E) showed improved pulmonary consolidation; liver function remained normal. Levofloxacin was discontinued, and linezolid was converted from intravenous to oral administration. By day 43, the infant's cough had nearly resolved; complete blood count and liver enzymes were within normal ranges. A four-drug anti-TB regimen of isoniazid, rifampicin, linezolid, and ethambutol was continued, and methylprednisolone was transitioned to oral prednisone until discharge.

On day 48, liver function remained stable (Figures 2G,H), and the patient was discharged with instructions for continued oral four-drug anti-TB therapy and hepatoprotective treatment.

One-month post-discharge, follow-up chest CT showed significant lesion resolution, with normal liver function and hematological parameters. The total planned duration of anti-TB therapy is six months.

## Discussion

This case represented the first reported instance of an infant co-infected with both pulmonary TB and pertussis—two distinct and highly infectious diseases. The overlapping clinical manifestations in such co-infection can easily lead clinicians to misdiagnose the condition as severe bacterial pneumonia, particularly in the absence of routine screening for TB and pertussis. Pediatric tuberculosis often presents with nonspecific symptoms, especially in neonates, who may exhibit only failure to thrive or feeding difficulties. Radiographically, it may mimic pneumonia or pulmonary consolidation, further resembling a typical bacterial infection. In contrast, pertussis is characterized by persistent paroxysmal coughing, often without radiological abnormalities. When both diseases are present in a single patient, the clinical picture becomes even more atypical.

In our case, the infant presented with cough and fever, accompanied by respiratory distress and hypoxia. Imaging revealed extensive pulmonary consolidation, and laboratory findings showed marked leukocytosis dominated by neutrophils with significantly elevated CRP levels, features typically associated with severe bacterial infection. Considering the infant's geographic and epidemiologic background of residing in a TB-endemic area with high pertussis prevalence among young infants, we included both pathogens in the diagnostic workup and ultimately confirmed the dual infection.

Therapeutically, managing co-infection with TB and pertussis in a neonate presents considerable challenges. According to the latest 2024 clinical guidelines for pertussis prevention and treatment, macrolides remain the first-line therapy. However, macrolide resistance rates in China are alarmingly high (70%–100%) (9). In our patient, the lack of clinical improvement after three days of erythromycin raised suspicion of resistance. For macrolide-resistant pertussis, sulfamethoxazole-trimethoprim is the preferred alternative for children over two months of age, while cefoperazone-sulbactam or piperacillin-tazobactam is recommended for younger infants (9). Levofloxacin is considered a secondary option. Given the patient's critical condition, severe pulmonary infection, and concurrent TB, along with the fact that levofloxacin is a second-line anti-TB agent, we selected it after thorough discussion with the family. The clinical response confirmed its efficacy in treating both infections.

Furthermore, due to the immaturity of hepatic metabolism in neonates and the known hepatotoxicity of *M. tuberculosis*, drug-induced liver injury (DILI) is a major concern during TB treatment. In children, the incidence of DILI can reach 65%, particularly in cases of severe hepatic damage, which can compromise treatment outcomes and increase mortality risk (10). In this case, standard WHO-recommended first-line therapy (HRZ: isoniazid, rifampicin, pyrazinamide) (11) was initiated but rapidly led to elevated liver enzymes and jaundice. Sequential discontinuation of rifampicin, pyrazinamide, and isoniazid was necessary. Linezolid, known for its utility in severe TB cases, was introduced as an alternative. Once hepatic function normalized, the patient was transitioned to a regimen of isoniazid, rifampicin, ethambutol, and oral linezolid, with liver enzymes remaining within normal limits. Continuous liver function monitoring throughout the course enabled timely detection and management of hepatotoxicity, contributing significantly to the success of TB therapy.

In summary, this case underscores the importance of maintaining a high index of suspicion for atypical pathogens such as *M. tuberculosis* and *B. pertussis* in neonates presenting with unexplained severe pneumonia, especially in endemic areas. Routine screening for these pathogens should be considered. Therapeutic management must be individualized, with careful consideration of antibiotic resistance patterns and potential drug toxicities, particularly hepatic injury. Dynamic assessment and close monitoring of adverse drug reactions are essential. This successful outcome provides valuable insights for the management of similarly complex pediatric infections in the future.

## Data Availability

The original contributions presented in the study are included in the article/Supplementary Material, further inquiries can be directed to the corresponding authors.
